# Baron Desgenettes

**Published:** 1838-07

**Authors:** 


### BARON DESGENETTES.

René Dufriche Desgenettes was born at Alençon, in 1762. He was descended from an old family, whose members have for several centuries illustrated the magistracy and the bar of that city. His earliest instruction was received at Alençon, but he was soon transferred to Paris, where he studied at the College of St. Barbe. At the age of eighteen, he made choice of the medical profession; and, after having attended the principal schools of Paris, he visited those of England and Italy. He has recorded the remarkable events of this period of his life in his "Souvenirs," a book which was the production of his latter years, and which shews the extent and variety of information which he had acquired by reading, observation, and the intercourse which he had enjoyed with the first men of his time. Returning to France in 1789, he received his degree of doctor at Montpellier, where he enjoyed the friendship of Fouquet and Barthez. He wrote his thesis on the Lymphatic System; a subject which, being then entirely new, attracted universal attention; and he demonstrated the anatomy of the absorbents from drawings, which he had received from his friend Mascagni. In 1793, he was attached professionally to the army of Italy, and from that time he continued to follow the fortunes of Napoleon to the close of his career. His celebrated experiments on the contagious nature of the Plague are well known; but it was not less his great professional talents than his unbending integrity which recommended him to the Emperor, who could appreciate his upright and elevated mind, which never stooped to low flattery or cowardly complaisance. He directed his attention particularly to the hygienic branch of military medicine, and it was this portion of the science which he professed at the School of Medicine in Paris. Political events deprived him of his professorship, which was restored to him after the revolution of July; but the caprices of fortune could not take from him that just consideration which was his due, nor that peace of mind and self-respect which his conduct had procured for him.*

* Eloge par M. Pariset. Bulletin de l'Academie, 28 Feb. 1837.

